# P-1360. Activity of Cefiderocol against Carbapenem Non-susceptible Enterobacterales, Including Molecularly Characterized Multidrug-resistant Clinical Isolates, Causing Infections in United States Hospitals (2020–2023)

**DOI:** 10.1093/ofid/ofae631.1537

**Published:** 2025-01-29

**Authors:** Rodrigo E Mendes, Cory Hubler, Danielle Beekman, Joshua Maher, Helio S Sader, Mariana Castanheira

**Affiliations:** JMI Laboratories, North Liberty, Iowa; Element Materials Technology/Jones Microbiology Institute, North Liberty, Iowa; Element Materials Technology/Jones Microbiology Institute, North Liberty, Iowa; Element Materials Technology/Jones Microbiology Institute, North Liberty, Iowa; JMI Laboratories, North Liberty, Iowa; JMI Laboratories, North Liberty, Iowa

## Abstract

**Background:**

Cefiderocol (FDC) is a siderophore cephalosporin that hijacks the iron transport system of Gram-negative bacteria to facilitate cell entry. FDC remains stable to hydrolysis in the presence of serine β-lactamases (BL) (e.g. ESBLs, KPC, and OXA-type carbapenemases) and metallo-BL (MBL). FDC and comparator activities were analyzed against carbapenem-nonsusceptible (nonS) Enterobacterales (ENT), including molecularly characterized isolates, collected as part of the SENTRY Antimicrobial Surveillance Program for the US.

**Figure:**
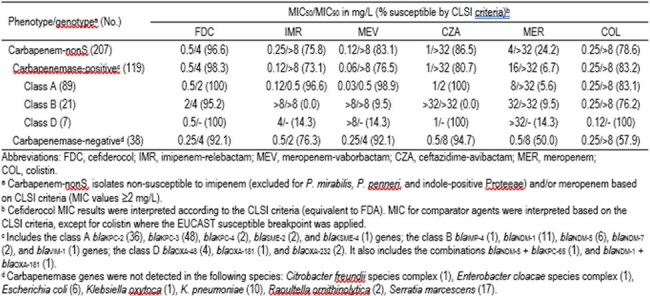

**Methods:**

15,147 ENT were collected from 35 sites in 2020–2023. Susceptibility (S) testing was performed by broth microdilution with cation-adjusted Mueller-Hinton broth (CAMHB) for comparators and iron-depleted CAMHB for FDC. CLSI criteria (same as the FDA) were used for FDC; whereas CLSI were applied for comparators, except for colistin (EUCAST). ENT with MIC ≥2 mg/L (nonS by CLSI) for imipenem or meropenem (MER) were screened for BL genes.

**Results:**

1.4% (207/15,147) isolates were carbapenems-nonS, and among these 57.5% (119/207) carried carbapenemases. Class A *bla*_KPC_ (72.3%; 86/119) prevailed, followed by class B MBL genes (17.6%; 21/119) and class D *bla*_OXA-48_ variants (5.9%; 7/119). FDC (96.6–98.3%S) had MIC_50_ of 0.5 mg/L and MIC_90_ of 4 mg/L against carbapenem-nonS and carbapenemase-carrying isolates; other agents had S of 6.7–86.5% (Table). FDC (100%S) and β-lactam-β-lactamase inhibitor (BL/BLI) combinations (96.6–100%S) were active against ENT carrying class A carbapenemases, whereas only FDC (MIC_50/90_, 2/4 mg/L; 95.2%S) had activity against those carrying class B genes. FDC and ceftazidime-avibactam (CAZ-AVI) were active (100%S for both) against ENT carrying class D genes. FDC (MIC_50/90_, 0.25/4 mg/L; 92.1%S), MER-vaborbactam (MIC_50/90_, 0.25/4 mg/L; 92.1%S) and CAZ-AVI (MIC_50/90_, 0.5/8 mg/L; 94.7%S) were most active against carbapenem-nonS, but carbapenemase-negative ENT.

**Conclusion:**

FDC activity against carbapenem-nonS ENT collected in the US. FDC activity was consistent across different genotypes, including against isolates carrying carbapenemase genes, where approved BL/BLI combinations showed limited activity. These data demonstrate FDC as an important option for the treatment of infections caused by carbapenem-nonS ENT.

**Disclosures:**

**Rodrigo E. Mendes, PhD**, GSK: Grant/Research Support

